# Comparative binding and activity analysis of known serine hydroxymethyltransferase inhibitors in biochemical assays

**DOI:** 10.1039/d5ra08513f

**Published:** 2026-07-02

**Authors:** Julian Gräb, Christine Wagner, Charlotte Beber, Jennifer Szczesny, Stefan Rubner, Ioannis Papasotiriou

**Affiliations:** a Research Genetic Cancer Centre Central Europe GmbH Weinbergweg 22 06120 Halle (Saale) Germany research@rgcc-centraleurope.com; b Research Genetic Cancer Centre International GmbH Baarerstrasse 95 6300 Zug Switzerland

## Abstract

Serine hydroxymethyltransferase (SHMT) is a crucial enzyme in folate metabolism, transferring a methylene group from serine to tetrahydrofolate (THF). It plays a key role in one-carbon metabolism, particularly in purine and thymidine biosynthesis, which has been linked to various cancers. Recently, a small number of small-molecule inhibitors distinct from traditional antifolates have been identified, such as SHIN1, SHIN2, AGF347 and W478. However, comprehensive cell-free biochemical data for binding and activity analysis of these inhibitors are lacking. By using thoroughly characterized recombinant SHMT1/2, this study investigates direct inhibitor binding by protein thermal shift assay and aims to expand the use of a known biochemical coupled enzyme approach, which has not yet been applied to determine the inhibitory activity of chemical test compounds, to evaluate the activity of these inhibitors against SHMT1 and SHMT2. Our work provides a comprehensive analysis of nine different SHMT1/2 inhibitors, thereby presenting a new methodological strategy that fills a gap in the existing literature on SHMT inhibition as well as opening up new avenues for the development and investigation of SHMT1/2 inhibitors.

## Abstract 2

This study presents a comparative biochemical characterization of known SHMT inhibitors and thereby introduces a new methodological strategy for a cost-effective coupled enzymatic assay to analyze SHMT inhibition.

## Graphical abstract

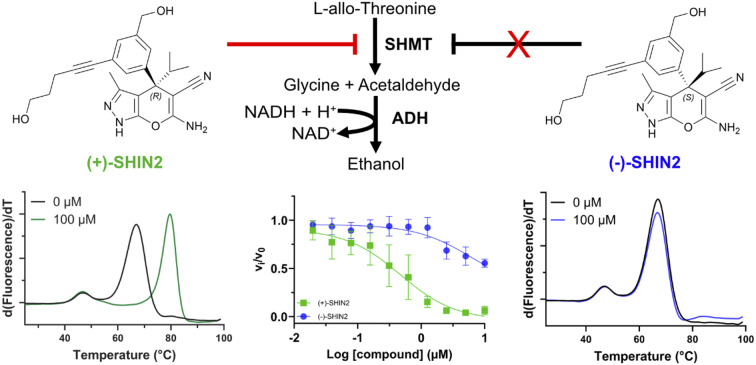

## 1 Introduction

SHMT is a pyridoxal phosphate (PLP) dependent enzyme that plays an important role in the folate metabolism by catalyzing the transfer of a methylene group from serine to tetrahydrofolate (THF). SHMT, therefore, has a crucial function in one-carbon metabolism, particularly in the biosynthesis of purines and thymidine. In human cells, there are two distinct SHMT proteins: cytosolic SHMT1 and mitochondrial SHMT2, which are encoded by two different genes.^1^

Enzymes involved in folate-mediated one-carbon metabolism are frequently dysregulated in human cancers.^2,3^ Consequently, extensive efforts have been made to target these enzymes with small-molecule inhibitors. Particularly for the folate cycle enzyme dihydrofolate reductase (DHFR), numerous compounds have entered preclinical phases.^4^ SHMT1 and SHMT2 have also been associated with various human cancers, including lung cancer, leukemia, and glioblastoma.^5–8^ Early SHMT inhibitors were antifolates; however, these compounds often exhibited poor metabolic stability, toxic side effects, or displayed only moderate activity.^9,10^ In recent years, several small-molecule inhibitors of SHMT1/2 have been identified that are structurally distinct from antifolates. Many of these inhibitors, particularly pyrazolopyrans as SHIN1, SHIN2, and derivatives as well as AGF347, Hit 1 and W478 ,^7,11–15^ have been studied with available *in vitro* and *in vivo* data. However, in the case of the most active small-molecule SHMT inhibitor, (+)-SHIN2 and its much less active enantiomer (−)-SHIN2, comprehensive biochemical inhibition data are currently lacking.

In this study, we describe the application of a biochemical assay, previously used exclusively to analyze the enzymatic activity of SHMT1, to determine the activity of SHMT1/2 inhibitors. In particular, we use this assay to provide a comparative activity analysis of nine selected SHMT1/2 inhibitors. Thus, we extend the methodology of analyzing the activity of SHMT inhibitors and provide the missing biochemical data for SHIN2. In addition, we provide the first direct binding data as determined in protein thermal shift assays for most of these compounds.

## 2 Results and discussion

### 2.1 The principle of an ADH-coupled enzymatic assay to monitor SHMT activity and inhibition by small molecules

The most commonly used biochemical assay to determine the enzymatic activity of SHMT1/2 is an absorbance-based, coupled enzymatic assay using the enzyme methylene tetrahydrofolate dehydrogenase (MTHFD),^16,17^ which has also been described for the analysis of the activity of inhibitors. Other methods are also described including mass spectrometry- and liquid chromatography-based measurements,^7,18^ a recently developed fluorescence-based assay,^12^ and additional absorbance-based methods.^19^ The latter include an assay in which the SHMT-catalyzed, THF-independent retro-aldol cleavage of threonine is coupled to the reaction catalyzed by the enzyme alcohol dehydrogenase (ADH). In this assay, threonine is converted into acetaldehyde, which is subsequently reduced to ethanol by ADH, and NADH is consumed as a co-substrate.^16,18,20^ The reaction is readily detectable by a decrease in NADH absorbance. Currently, this assay is used to measure the enzymatic activity of SHMT1/2 but has not yet been applied to investigate SHMT1/2 inhibitors.

Since the ADH-coupled assay is a simple, absorbance-based biochemical method independent of any fluorescent probe, which would have to be accessible, but instead based on standard chemicals readily available, we intended to expand its application, thereby presenting a new methodological strategy for the investigation of SHMT1/2 inhibitors.

Thereby we aimed to apply nine described and commercially available small-molecule inhibitors including pyrazolopyrans as SHIN1, SHIN2, and derivatives as well as AGF347, Hit 1 and W478 (Table S1).

### 2.2 Binding of inhibitors to purified SHMT1 and SHMT2

We cloned, recombinantly expressed, and purified human SHMT1 and SHMT2 from bacteria and characterized both proteins in light scattering and enzymatic activity assays (Table S2). The obtained specific activities for SHMT1 (As = 0.46 ± 0.01 U mg^−1^) and SHMT2 (As = 0.42 ± 0.01 U mg^−1^) demonstrated that the proteins are applicable for our assay setup (Table S2). The *K*_m_ values of l-allo-threonine for SHMT1 (1.22 ± 0.40 mM) and SHMT2 (3.99 ± 1.03 mM) were consistent with published values (Fig. S1).^20^

**Fig. 1:**
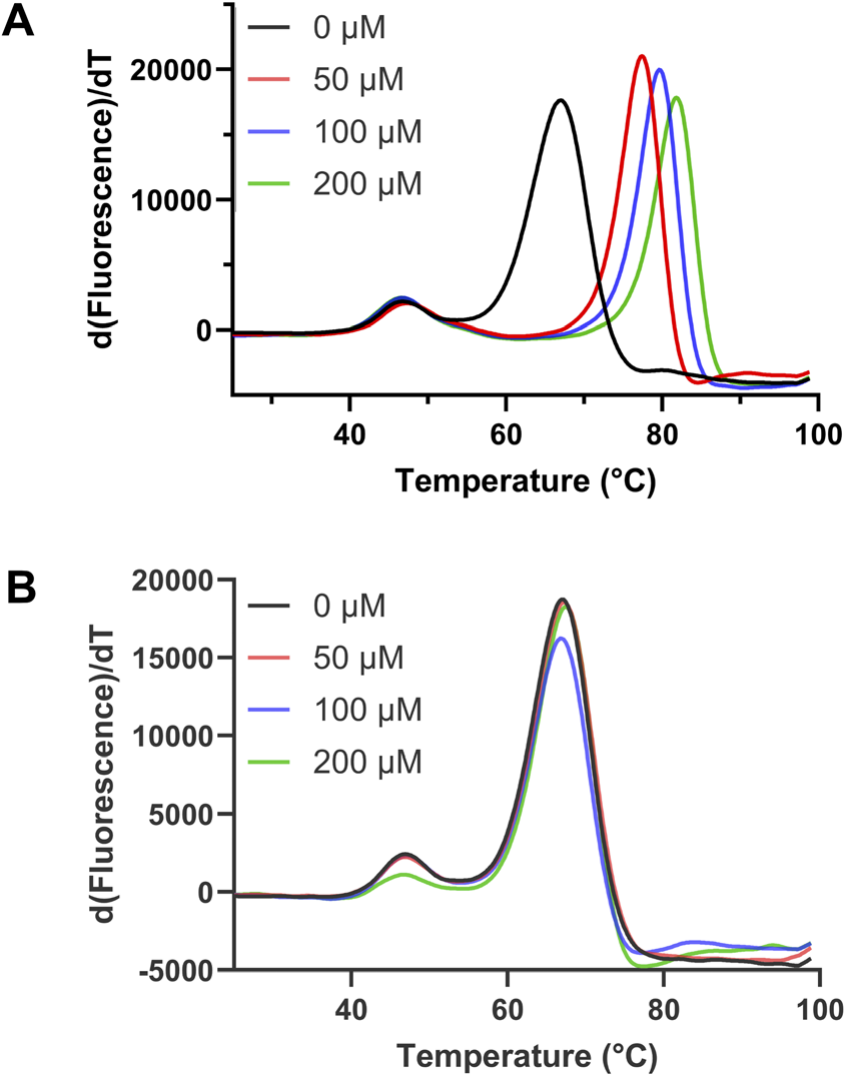
Binding of (A) (+)-SHIN2 and (B) (−)-SHIN2 to SHMT1 as analyzed in protein thermal shift assays. The first derivative of the fluorescence intensity over temperature is plotted against the temperature. All experiments were performed in triplicate.

Then, we analyzed the binding of the nine compounds to SHMT1 and SHMT2 in protein thermal shift assays (TSA, Table 1, Fig. 1, S2 and S3). The SHIN1/2 isomers (+)-SHIN1 and (+)-SHIN2, which are described to be the active isomers, displayed Δ*T*_m_ values of 16.5 ± 0.8 °C and 13.1 ± 0.2 °C, respectively, indicating strong binding. In contrast, (−)-SHIN1 showed no binding to SHMT1 (Δ*T*_m_ = −0.17 ± 0.19 °C), whereas (−)-SHIN2 displayed very weak binding (Δ*T*_m_ = 0.72 ± 0.36 °C) as compared with (+)-SHIN1/2.

**Table 1:** Binding of compounds to SHMT1 and SHMT2 as determined in thermal shift assays. Δ*T*_m_ are indicated at 100 µM inhibitor concentration. All experiments were carried out at least in triplicate (mean values ± standard deviations)

Compound	SHMT1 Δ*T*_m_ (°C)	SHMT2 Δ*T*_m_ (°C)
(+)-SHIN1	16.5 ± 0.8	19.4 ± 1.7
(−)-SHIN1	−0.17 ± 0.19	−0.44 ± 2.35
(+)-SHIN2	13.1 ± 0.2	18.1 ± 0.5
(−)-SHIN2	0.72 ± 0.36	0.22 ± 0.86
AGF347	1.38 ± 0.24	2.59 ± 1.38
Hit 1	2.61 ± 0.04	10.1 ± 0.3
W478	−0.40 ± 0.67	4.51 ± 0.63
SHMT-IN-1	5.53 ± 0.15	3.58 ± 0.24
SHMT-IN-2	16.1 ± 0.2	16.3 ± 0.1

The pyrazolopyrans SHMT-IN-1 (Δ*T*_m_ = 5.53 ± 0.15 °C) and SHMT-IN-2 (Δ*T*_m_ = 16.1 ± 0.2 °C), which are based on the same chemical scaffold as SHIN1 and SHIN2, showed moderate to strong binding. AGF347 (Δ*T*_m_ = 1.38 ± 0.24 °C) and Hit 1 (Δ*T*_m_ = 2.61 ± 0.04 °C) showed slight binding to SHMT1. All compounds showed similar binding to SHMT2, with the melting temperature shift Δ*T*_m_ of SHMT2 comparable to SHMT1, except Hit 1 and W478, which displayed stronger binding to SHMT2. W478 showed binding exclusively to SHMT2, but not SHMT1 (Table 1). However, we observed denaturation of SHMT1 and SHMT2 at room temperature, which was indicated by discolouration of the solution in the well and broadened peaks in the melting curve (Fig. S2 and S3).

The summarized activity data for our purified SHMT1 and SHMT2, along with compound binding results from thermal shift assays, enabled the application of our SHMT1 and SHMT2 in the ADH-coupled assay to determine the inhibitory potential of the selected inhibitors, with the exception of W478, for which the results at the highest test concentration should at least be treated with caution.

### 2.3 THF-independent retro-aldol cleavage by SHMT1/2

**Fig. 2:**
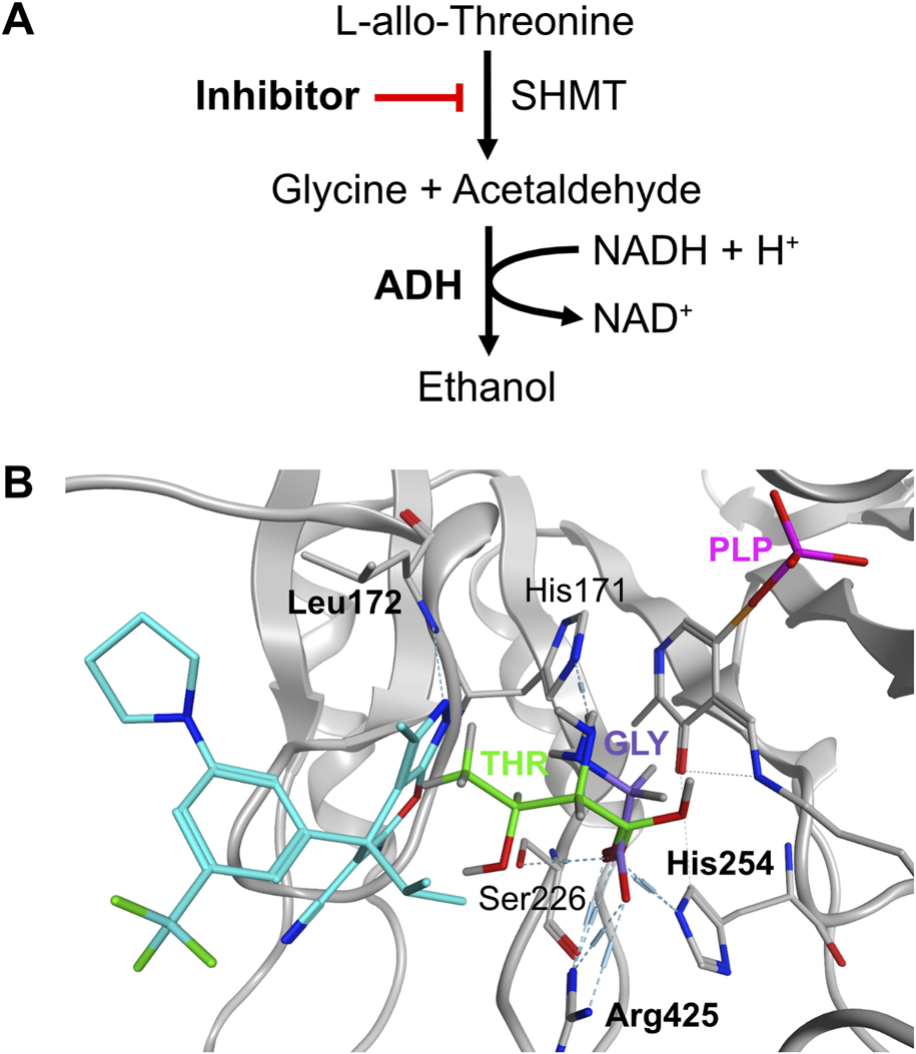
(A) Principle of the ADH-coupled inhibition assay. (B) Docking pose of l-allo-threonine (green) at the glycine binding site in the crystal structure of SHMT2 (PDB: 5V7I) complexed with glycine (carbon atoms in purple), a folate-competitive pyrazolopyran inhibitor (carbon atoms in cyan), and pyridoxal phosphate (PLP; phosphate group in magenta).

Since we assume that the compounds tested in this study act as THF competitive compounds^7,11,13–15^ and the retro-aldol cleavage of threonine by SHMT1/2 is independent from THF as a co-substrate, we next aimed to rationalize the applicability of our assay for the investigation of these inhibitors (Fig. 2A).

To this end, we docked l-allo-threonine into the crystal structure of human SHMT2 (PDB: 5V7I) complexed with glycine and a folate-competitive pyrazolopyran inhibitor.

The docking predicted interactions of the carboxylic acid group of threonine with Arg425 and His254 by hydrogen bonds, which correspond to the same interactions that glycine forms with the protein in the crystal structure, making the docking pose appear plausible (Fig. 2B). The overlayed docking illustrates that threonine can sterically clash with the inhibitor preventing the formation of a hydrogen bond between the inhibitor and Leu172 of the protein, which could affect the binding of the inhibitor. It is therefore plausible that an assay based on THF-independent retro-aldol cleavage by SHMT1/2 could still be used to investigate THF-competitive inhibitors.

### 2.4 Application of the ADH-coupled enzymatic assay to monitor SHMT inhibition

To first proof that the inhibitors do not lead to misleading results due to their absorption characteristics or potential inhibition of the coupling enzyme ADH, we performed counter assays by carrying out an absorption wavelength scan, protein thermal shift assays and determined the enzymatic activity of ADH in the presence of the inhibitors.

The compounds did not display significant absorbance at the test wavelength of 340 nm, except W478 that showed slight absorbance at the test wavelength and an absorbance peak at 280 nm (Fig. S4), where its activity against SHMT2 was originally be determined.^15^ None of the compounds displayed relevant binding to ADH in thermal shift assays (Fig. S5 and Table S3) or reduced its enzymatic activity (Fig. S6 and Table S3).

Finally, we performed the ADH-coupled assay in the presence of the inhibitors. For the inhibition assays we converted the determined IC_50_ values into *K*_i_ values for a better comparability of the inhibitor activities using the Cheng–Prusoff equation.^21^ The equation assumes a competitive inhibition mode. We have not clearly shown competitive inhibition in a structural or kinetic investigation; however, we rationalized a competitive inhibition mode by molecular docking for the pyrazolopyran moiety (Fig. 2B), which most of our tested compounds carry. Also, the testing of inactive enantiomers of the inhibitors SHIN1 and SHIN2, which, in their active forms, are known to act as folate competitors, indicate a competitive reaction mode.

**Fig. 3:**
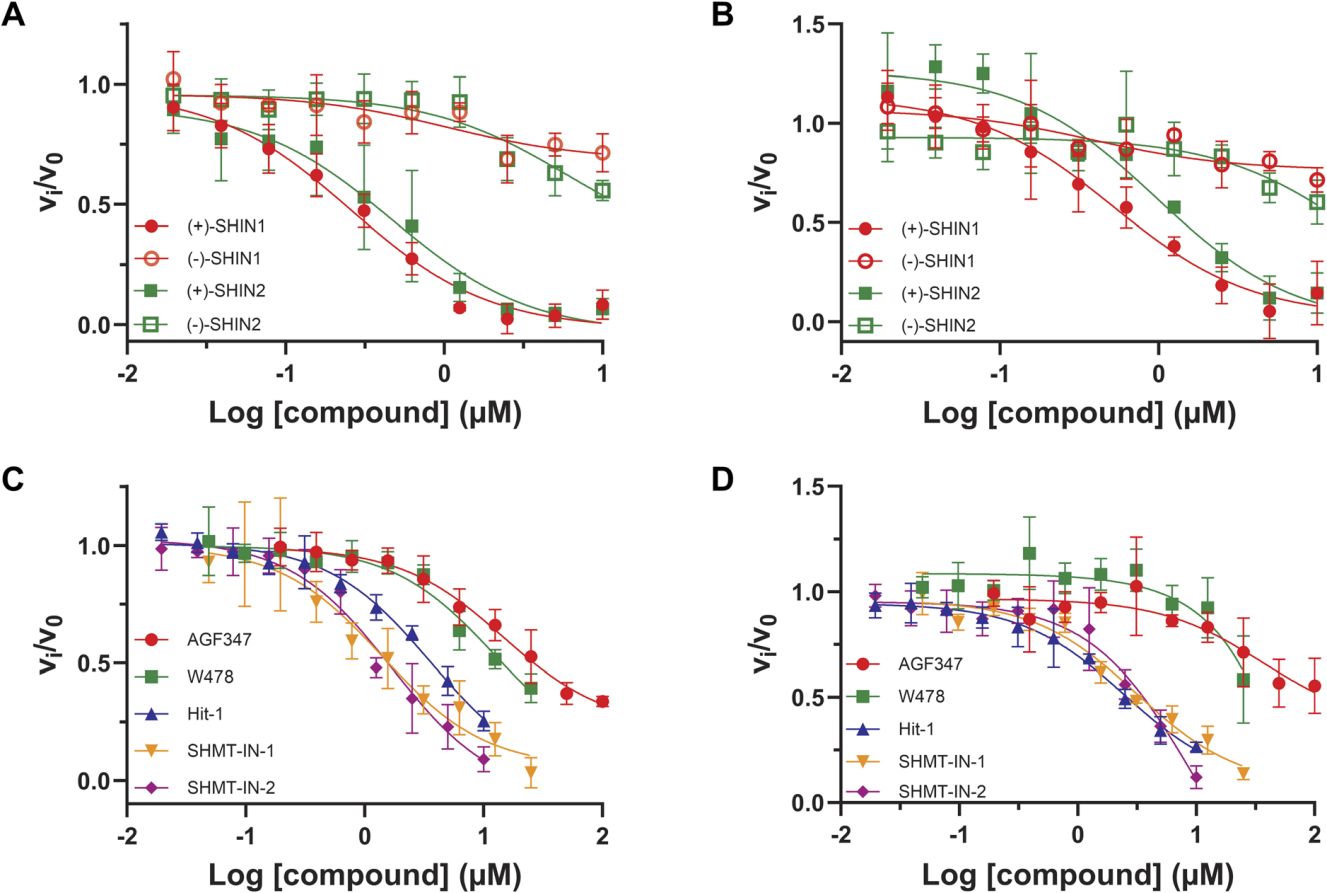
Activity of selected SHMT inhibitors in the ADH-coupled enzymatic assay. (A) Activity of SHIN1 and SHIN2 enantiomers against SHMT1. (B) Activity of SHIN1 and SHIN2 enantiomers against SHMT2. (C) Activity of inhibitors against SHMT1. (D) Activity of inhibitors against SHMT2. Initial reaction rates *v*_*i*_ of compound-containing reactions normalized against the initial reaction rates *v*_0_ of the control reactions are plotted against the compound concentrations. All experiments were performed at least in triplicate. Error bars represent standard deviations.

Also docking of AGF347 by Dekhne *et al.* as well as docking of W478 into the serine binding site carried out by Han and co-workers proposed a competitive inhibition mechanism.^15,22^ Hit 1 was described to act *via* a non-competitive mechanism by Nonaka *et al.* excluding the usage of the Cheng–Prusoff equation for this compound.^12^

(+)-SHIN1 displayed activities against SHMT1 (*K*_i_ = 0.09 ± 0.02 µM) and SHMT2 (*K*_i_ = 0.47 ± 0.17 µM) with inhibitory constants in the submicromolar to nanomolar range and a slight selectivity for SHMT1 over SHMT2 (Fig. 3A, B and Table 2). (+)-SHIN2, which was described as more potent than (+)-SHIN1 in cell-based assays,^11^ was slightly more active against SHMT1 (*K*_i_ = 0.15 ± 0.08 µM) as compared to SHMT2 (*K*_i_ = 0.97 ± 0.09 µM) with *K*_i_ values comparable to (+)-SHIN1 in our assay. The respective stereoisomers, (−)-SHIN1 and (−)-SHIN2, previously reported to be less active,^7,11^ exhibited markedly reduced activities in the double-digit micromolar range to both enzymes (Fig. 3A, B and Table 2). These data are consistent with the binding data from TSA demonstrating that the compounds bind isomer-specifically (Table 1).

**Table 2:** IC_50_ and *K*_i_ values for the inhibition of SHMT1 and SHMT2 by selected inhibitors as determined in the ADH-coupled assay. All experiments were carried out at least in triplicate (mean values ± standard deviations). Apparent *K*_m_ values for l-allo threonine are 1.22 ± 0.40 mM (SHMT1) and 3.99 ± 1.03 mM (SHMT2). n/a-not applicable

Compound	SHMT1 IC_50_ (µM) or inhibition (%) at the indicated concentration	SHMT1 *K*_i_ (µM)	SHMT2 IC_50_ (µM) or inhibition (%) at the indicated concentration	SHMT2 *K*_i_ (µM)
(+)-SHIN1	0.23 ± 0.05	0.09 ± 0.02	0.70 ± 0.25	0.47 ± 0.17
(−)-SHIN1	28.5 ± 7.9% at 10 µM	n/a	28.5 ± 6.2% at 10 µM	n/a
(+)-SHIN2	0.40 ± 0.22	0.15 ± 0.08	1.45 ± 0.14	0.97 ± 0.09
(−)-SHIN2	44.2 ± 4.2% at 10 µM	n/a	39.7 ± 11% at 10 µM	n/a
AGF347	26.9 ± 7.5	10.2 ± 2.8	44.6 ± 13.1% at 100 µM	n/a
Hit 1	3.67 ± 0.43	n/a	2.58 ± 0.27	n/a
W478	14.50 ± 2.74	5.50 ± 1.04	42 ± 21% at 25 µM	n/a
SHMT-IN-1	1.60 ± 0.51	0.61 ± 0.19	3.43 ± 0.51	2.29 ± 0.34
SHMT-IN-2	1.82 ± 0.56	0.63 ± 0.21	3.76 ± 0.93	2.36 ± 0.58

The other two pyrazolopyrans, SHMT-IN-1 and SHMT-IN-2, were marginally less active than SHIN1 and SHIN2, which is consistent with the activities originally reported.^7,14^ They exhibited similar efficacy when compared with one another, with *K*_i_ values for SHMT1 in the sub-micromolar range and a slight preference for SHMT1 over SHMT2 being observed (Fig. 3C, D and Table 2). Hit 1 was moderately active against SHMT1 (IC_50_ = 3.67 ± 0.43 µM) and SHMT2 (IC_50_ = 2.58 ± 0.27 µM) and displayed no selectivity, which was consistent with published activities.^12^ AGF347 showed moderate activity against SHMT1 (*K*_i_ = 10.2 ± 2.8 µM), but markedly reduced activity against SHMT2 (44.6 ± 13.1% inhibition at 100 µM, the highest concentration tested). W478 displayed moderate activity against SHMT1 (*K*_i_ = 5.50 ± 1.04 µM), but only negligible activity against SHMT2 (42 ± 21% at 25 µM, the highest concentration tested) in the context of our assay (Fig. 3C, D and Table 2).

Further, we determined *Z*′ factors for both inhibitory assays. While the *Z*′ factor for our SHMT1 assay (*Z*′ = 0.52 ± 0.04) indicated its suitability for future high-throughput screening applications for the discovery of new SHMT inhibitors, the value for our SHMT2 assay (*Z*′ = 0.36 ± 0.05) was relatively low for this sort of biochemical assay demonstrating that the assay is close to the lower border of suitability for HTS (Fig. S7). Nevertheless, the *Z*′ factor for SHMT2 suggests that the assay is potentially still suitable for HTS, although to a lower extent than our SHMT1 assay. Improvement of protein production or changes of the buffer composition in order to reduce heterogeneity of the SHMT2 as well as SHMT1 preparation might lead to an increase of the *Z*′ factor for both enzymes.

## 3 Conclusions

In summary, we demonstrate the first application of the alcohol dehydrogenase-coupled enzymatic assay for the determination of the activity of small-molecule SHMT1/2 inhibitors. In addition, we provide the first cell-free biochemical data for the activity of (+)-SHIN2 and (−)-SHIN2, as well as the first direct binding data from thermal shift assays for SHIN1, SHIN2, SHMT-IN-1, SHMT-IN-2, AGF347 and W478.

The ADH-coupled inhibition assay is a reproducible assay that takes advantage of the coupling enzyme ADH, which can be expressed recombinantly in yeast with high yields. It is based on the application of standard chemicals rather than specific chemical probes, which makes this assay, using a simple instrumental setup, readily applicable. Importantly, this assay does not require THF as a co-substrate, thereby overcoming potential issues due to the oxygen sensitivity of THF. Our results expand the available methodologies for investigating SHMT1/2 inhibitors through a cost-effective, straightforward, reliable and high-throughput accessible approach and thereby contribute to the further development of new SHMT1/2 inhibitors.

## 4 Materials and methods

The general chemicals were purchased from Carl Roth GmbH + Co. KG (Karlsruhe, Germany), VWR International GmbH (Darmstadt, Germany), Merck KGaA (Darmstadt, Germany), and Thermo Fisher Scientific (Life Technologies GmbH, Darmstadt, Germany). The supplier for more specific consumables is stated in the method description itself below.

### 4.1 SHMT inhibitors

Inhibitors (MedChemExpress, USA) were purchased from Biozol GmbH (Germany, (+)-SHIN1: MCE-HY-112066A, (−)-SHIN1: MCE-HY-112066B, (+)-SHIN2: MCE-HY-134978A, (−)-SHIN2: MCE-HY-134978B, SHMT-IN-1: MCE-HY-129651, SHMT-IN-2: MCE-HY-129226, W478: MCE-HY-178432). AGF347 and Hit 1 were purchased from Merck (Sigma-Aldrich, AGF347: SML2904, Hit 1: SML2699).

### 4.2 Protein thermal shift assay

Protein thermal shift assays were performed applying protein thermal shift dye (Thermo Fisher Scientific, 4461146). Assays were carried out in 50 mM HEPES pH 7.5, 100 mM NaCl, 0.5 mM EDTA, 0.005% (v/v) Tween 20, 1 mM DTT and 4% (v/v) DMSO. SHMT proteins were applied at 2 µM. Alcohol dehydrogenase (ADH, Thermo Fisher Scientific, J65869.VEX) was applied at 2.4 µM. Compounds were applied at 50 µM, 100 µM and 200 µM. Proteins and compounds were incubated at room temperature for 40 minutes prior to the addition of the protein thermal shift dye. Assays were performed in transparent 384 well plates. Plates were centrifuged for 1 minute at 215×*g* prior the measurement. Melting curves from 25 °C to 99 °C with a temperature increase of 0.05 °C were recorded using a real time PCR cycler.

### 4.3 SHMT1/2 inhibition assay

All assays were carried out in assay buffer (50 mM HEPES pH 7.5, 100 mM NaCl, 0.5 mM EDTA, 0.005% (v/v) Tween 20 and 1 mM DTT) at room temperature. Reaction mixtures contained 0.5 µM SHMT1 or 1.5 µM SHMT2, 2 mM l-allo-threonine, 0.1 mM NADH, 10 U ADH and varying concentrations of test compounds (1.526 nM–100 µM, depending on the compound). The final DMSO concentration within the assay was 2%.

Compound in DMSO or DMSO (controls) and threonine were added to the wells and the reaction was started by the addition of a master mix composed of NADH, both enzymes (SHMT1 or SHMT2 and coupling ADH) and buffer using a multi-channel pipette. The absorbance at 340 nm was recorded in transparent 96-well plates with a total reaction volume of 100 µl per well. The initial reaction velocities *v*_*i*_ for each inhibitor concentration were determined by linear regression of the first 30 seconds and normalized against the initial reaction velocity of the non-inhibited reaction *v*_0_. *v*_*i*_/*v*_0_ values were plotted against the inhibitor concentration and the IC_50_ values were determined by applying a nonlinear four-parameter regression (Y=Bottom + (Top-Bottom)/(1 + 10^((LogIC50-X)*HillSlope))). IC_50_ values were converted into *K*_i_ values applying the Cheng–Prusoff equation (*K*_i_ = IC_50_/(1 + [*S*]/*K*_m_)).

### 4.4 Molecular docking

The crystal structure of human SHMT2 in complex with glycine, a folate-competitive pyrazolopyran inhibitor, and pyridoxal phosphate (PLP) (PDB ID: 5V7I; https://www.rcsb.org/structure/5V7I) was obtained from the RCSB Protein Data Bank. The ligand l-allo-threonine ((2*S*,3*S*)-2-amino-3-hydroxybutanoic acid (CID: 99289; https://pubchem.ncbi.nlm.nih.gov/compound/99289) was downloaded from PubChem.

All docking studies and visualizations were performed using the Molecular Operating Environment (MOE; Chemical Computing Group, version 2024; https://www.chemcomp.com). The structure was prepared using QuickPrep, which added missing hydrogens, optimized protonation states at pH 7.0, optimized side-chain conformations, and performed restrained energy minimization (Amber10:EHT force field) to relieve steric clashes while preserving the experimental geometry.

The native glycine ligand is present only in chain B; therefore, chain B was selected for docking. l-allo-threonine was docked (general docking method) into the same binding site without explicit removal of glycine, resulting in a visual overlap to illustrate potential steric competition.

The binding site was defined by the coordinates of the native glycine molecule. Docking was performed using the Triangle Matcher placement method with London dG scoring, followed by energy minimization under the default force field settings (Amber10:EHT). A total of twenty-six poses were generated to explore possible orientations of l-allo-threonine within the pocket. Docking parameters were as follows: placement method, Triangle Matcher; initial scoring, London dG; refinement, force field; and final rescoring, GBVI/WSA dG. The resulting poses were analyzed to assess steric hindrance and potential displacement of the folate-competitive pyrazolopyran inhibitor.

## Author contributions

Julian Gräb: investigation, methodology, formal analysis, data curation, visualization, writing – original draft; Christine Wagner: investigation, methodology, formal analysis, data curation, visualization, writing – review & editing; Charlotte Beber: investigation, writing – review & editing; Jennifer Szczesny: methodology, investigation, visualization, writing – review & editing; Stefan Rubner: supervision, project administration, conceptualization, methodology, writing – review & editing; Ioannis Papasotiriou: project administration, resources, writing – review & editing.

## Conflicts of interest

There are no conflicts to declare.

## Supplementary Material

RA-OLF-D5RA08513F-s001

## Data Availability

The data supporting this article have been included as part of the supplementary information (SI). To perform the docking study, publicly available data from RCSB Protein Data Bank (https://www.rcsb.org/structure/5V7I) for the crystal structure (PDB ID: 5V7I) and from PubChem (https://pubchem.ncbi.nlm.nih.gov/compound/99289) for the ligand (CID: 99289) were used. Supplementary information is available. See DOI: https://doi.org/10.1039/d5ra08513f.

## References

[cit1] Garrow T. A., Brenner A. A., Whitehead V. M., Chen X. N., Duncan R. G., Korenberg J. R., Shane B. (1993). J. Biol. Chem..

[cit2] Rosenzweig A., Blenis J., Gomes A. P. (2018). Front. Cell Dev. Biol..

[cit3] Locasale J. W. (2013). Nat. Rev. Cancer.

[cit4] Cuthbertson C. R., Arabzada Z., Bankhead A., Kyani A., Neamati N. (2021). ACS Pharmacol. Transl. Sci..

[cit5] Paone A., Marani M., Fiascarelli A., Rinaldo S., Giardina G., Contestabile R., Paiardini A., Cutruzzolà F. (2014). Cell Death Dis..

[cit6] Xie M., Pei D.-S. (2021). Invest. New Drugs.

[cit7] Ducker G. S., Ghergurovich J. M., Mainolfi N., Suri V., Jeong S. K., Hsin-Jung Li S., Friedman A., Manfredi M. G., Gitai Z., Kim H., Rabinowitz J. D. (2017). Proc. Natl. Acad. Sci..

[cit8] Minchenko O. H., Sliusar M. Y., Khita O. O., Minchenko D. O., Viletska Y. M., Halkin O. V., Levadna L. O., Cherednychenko A. A., Khikhlo Y. P. (2024). Endocr. Regul..

[cit9] Scaletti E., Jemth A., Helleday T., Stenmark P. (2019). FEBS Lett..

[cit10] Paiardini A., Fiascarelli A., Rinaldo S., Daidone F., Giardina G., Koes D. R., Parroni A., Montini G., Marani M., Paone A., McDermott L. A., Contestabile R., Cutruzzolà F. (2015). ChemMedChem.

[cit11] García-Cañaveras J. C., Lancho O., Ducker G. S., Ghergurovich J. M., Xu X., Da Silva-Diz V., Minuzzo S., Indraccolo S., Kim H., Herranz D., Rabinowitz J. D. (2021). Leukemia.

[cit12] Nonaka H., Nakanishi Y., Kuno S., Ota T., Mochidome K., Saito Y., Sugihara F., Takakusagi Y., Aoki I., Nagatoishi S., Tsumoto K., Sando S. (2019). Nat. Commun..

[cit13] Dekhne A. S., Ning C., Nayeen Md. J., Shah K., Kalpage H., Frühauf J., Wallace-Povirk A., O'Connor C., Hou Z., Kim S., Hüttemann M., Gangjee A., Matherly L. H. (2020). Mol. Pharmacol..

[cit14] Witschel M. C., Rottmann M., Schwab A., Leartsakulpanich U., Chitnumsub P., Seet M., Tonazzi S., Schwertz G., Stelzer F., Mietzner T., McNamara C., Thater F., Freymond C., Jaruwat A., Pinthong C., Riangrungroj P., Oufir M., Hamburger M., Mäser P., Sanz-Alonso L. M., Charman S., Wittlin S., Yuthavong Y., Chaiyen P., Diederich F. (2015). J. Med. Chem..

[cit15] Akram H. M. B., Liu Y., Dong J., Zhao X., Wang L., Zhao W., Liu H., Ma L., Han C. (2025). Bioorg. Chem..

[cit16] Angelaccio S., Chiaraluce R., Consalvi V., Buchenau B., Giangiacomo L., Bossa F., Contestabile R. (2003). J. Biol. Chem..

[cit17] Schirch L., Peterson D. (1980). J. Biol. Chem..

[cit18] Webb H. K., Matthews R. G. (1995). J. Biol. Chem..

[cit19] Luo D., Bai Z., Bai H., Liu N., Han J., Ma C., Wu D., Bai L., Li Z. (2024). J. Adv. Res..

[cit20] Ubonprasert S., Jaroensuk J., Pornthanakasem W., Kamonsutthipaijit N., Wongpituk P., Mee-udorn P., Rungrotmongkol T., Ketchart O., Chitnumsub P., Leartsakulpanich U., Chaiyen P., Maenpuen S. (2019). J. Biol. Chem..

[cit21] Yung-Chi C., Prusoff W. H. (1973). Biochem. Pharmacol..

[cit22] Dekhne A. S., Shah K., Ducker G. S., Katinas J. M., Wong-Roushar J., Nayeen Md. J., Doshi A., Ning C., Bao X., Frühauf J., Liu J., Wallace-Povirk A., O'Connor C., Dzinic S. H., White K., Kushner J., Kim S., Hüttemann M., Polin L., Rabinowitz J. D., Li J., Hou Z., Dann C. E., Gangjee A., Matherly L. H. (2019). Mol. Cancer Ther..

