# Supplementary material for: Comparative binding and activity analysis of known serine hydroxymethyltransferase inhibitors in biochemical assays
Source: RSC Adv. 2026 Jul 2;16(34):32101–6. doi: 10.1039/d5ra08513f (PMC13325798; doi:10.1039/d5ra08513f)
Supplement: RA-016-D5RA08513F-s001 [file RA-016-D5RA08513F-s001.pdf]

Supplementary Information (SI) for RSC Advances.
This journal is © The Royal Society of Chemistry 2026

Supplementary Information

Comparative binding and activity analysis of known serine hydroxymethyltransferase inhibitors in biochemical assays

Julian Gräb,†[a] Christine Wagner,†[a] Charlotte Beber,[a] Jennifer Szczesny,[a] Stefan Rubner*‡[a] and Ioannis Papasotiriou‡[b]

[a] *Research Genetic Cancer Centre Central Europe GmbH*
Weinbergweg 22, 06120 Halle (Saale), Germany
E-mail: research@rgcc-centraleurope.com
[b] *Research Genetic Cancer Centre International GmbH*
Baarerstrasse 95, 6300 Zug, Switzerland
† These first authors contributed equally
‡ These senior authors contributed equally

Table of Contents

Supplementary Figures 2

Supplementary Tables 8

Experimental Methods and Materials 11

Plasmid construction and recombinant protein expression 11

Protein purification 12

Static light scattering 12

Dynamic light scattering 12

Enzymatic activity assay 13

Determination of the K_m value of L-allo-threonine 13

Determination of the enzymatic activity of ADH in the presence of test compounds 13

Absorbance scan of test compounds 14

Z′ factor determination and calculation 14

Supplementary Figures

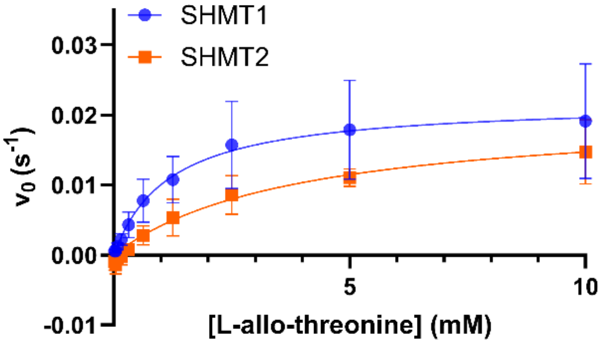

Figure S1: Retro-aldol cleavage of L-allo-threonine (0.0195 - 10 mM) for the determination of its K_m value for SHMT1 and SHMT2 as determined in the ADH-coupled assay. Initial reaction velocities were plotted against the L-allo-threonine concentration. All experiments were carried out in triplicate (mean values ± standard deviations).

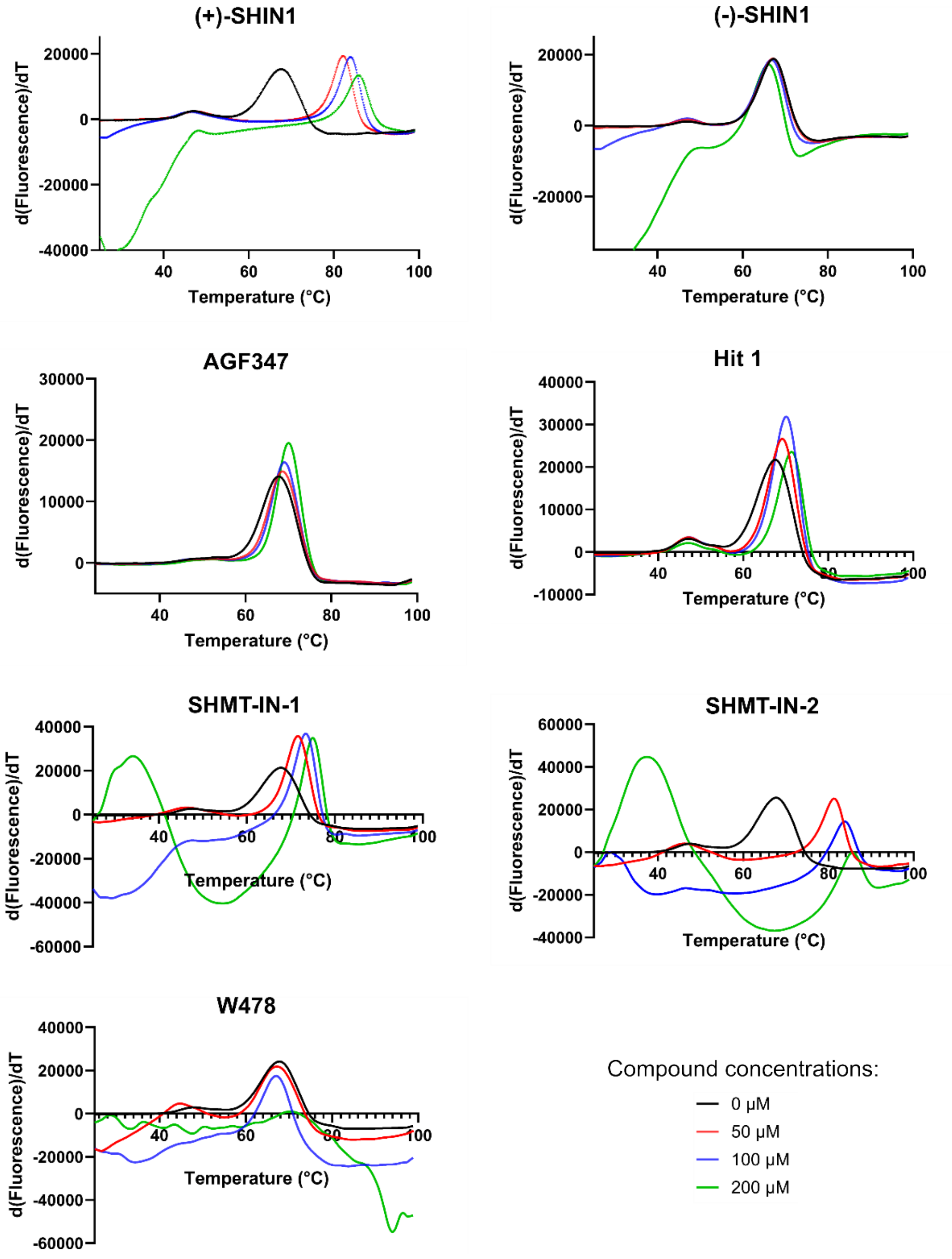

Figure S2: Binding of compounds to SHMT1 as analyzed in protein thermal shift assays. (+)-SHIN1, (-)-SHIN1, AGF347, Hit 1, SHMT-IN-1, SHMT-IN-2 and W478 were applied at compound concentrations of 0 µM (black), 50 µM (red), 100 µM (blue) and 200 µM (green). The curves for (+)-SHIN2 and (-)-SHIN2 are shown in Figure 1. All experiments were performed in triplicate.

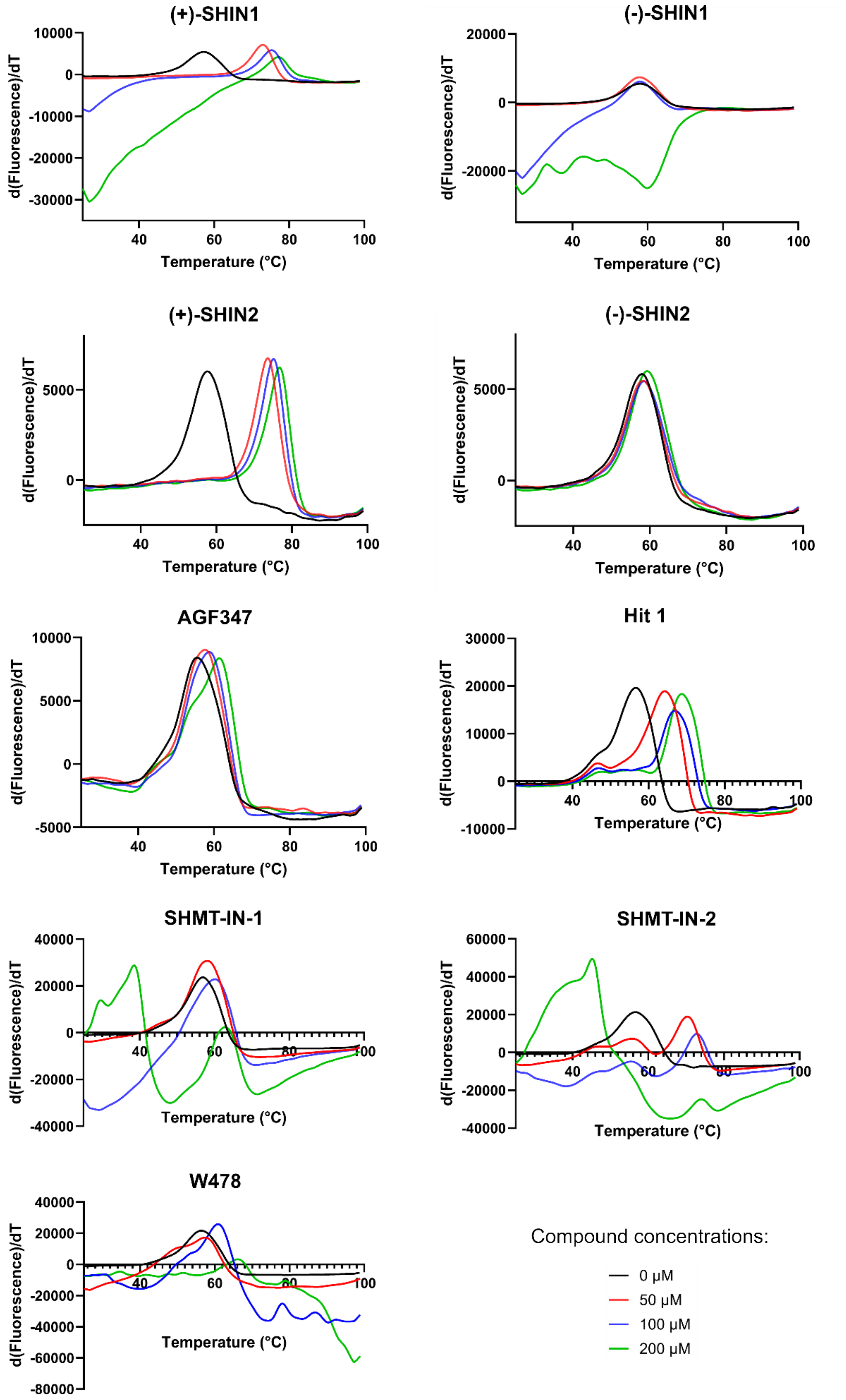

Figure S3: Binding of compounds to SHMT2 as analyzed in protein thermal shift assays. (+)-SHIN1, (-)-SHIN1, (+)-SHIN2, (-)-SHIN2, AGF347, Hit 1, SHMT-IN-1, SHMT-IN-2 and W478 were applied at compound concentrations of 0 µM (black), 50 µM (red), 100 µM (blue) and 200 µM (green). All experiments were performed in triplicate.

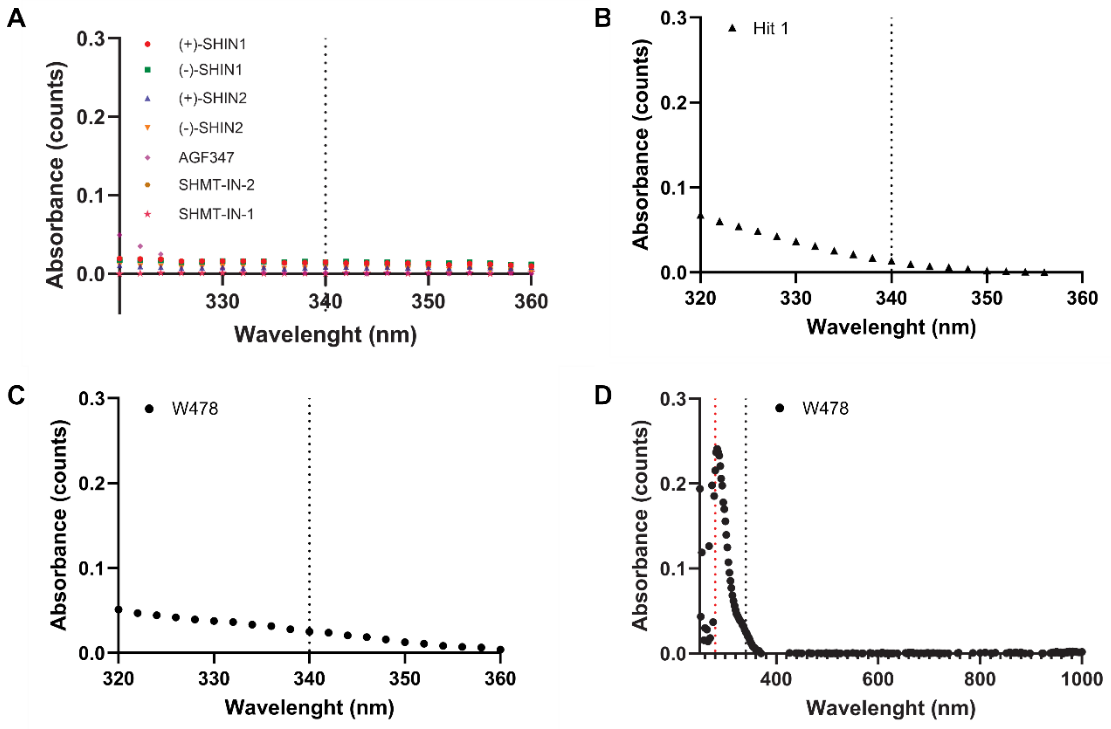

Figure S4: Absorbance scan of test compounds in SHMT inhibition assay buffer. (A) Inhibitors as listed, (B) Hit 1, and (C) W478 absorbance scan (excerpt 320 - 360 nm). (D) W478 absorbance scan (200 - 1000 nm, absorbance peak at 280 nm, red dotted line). The black dotted line marks the wavelength where the inhibition assay is recorded. The compound concentrations corresponded to the highest test concentration in the inhibition assays.

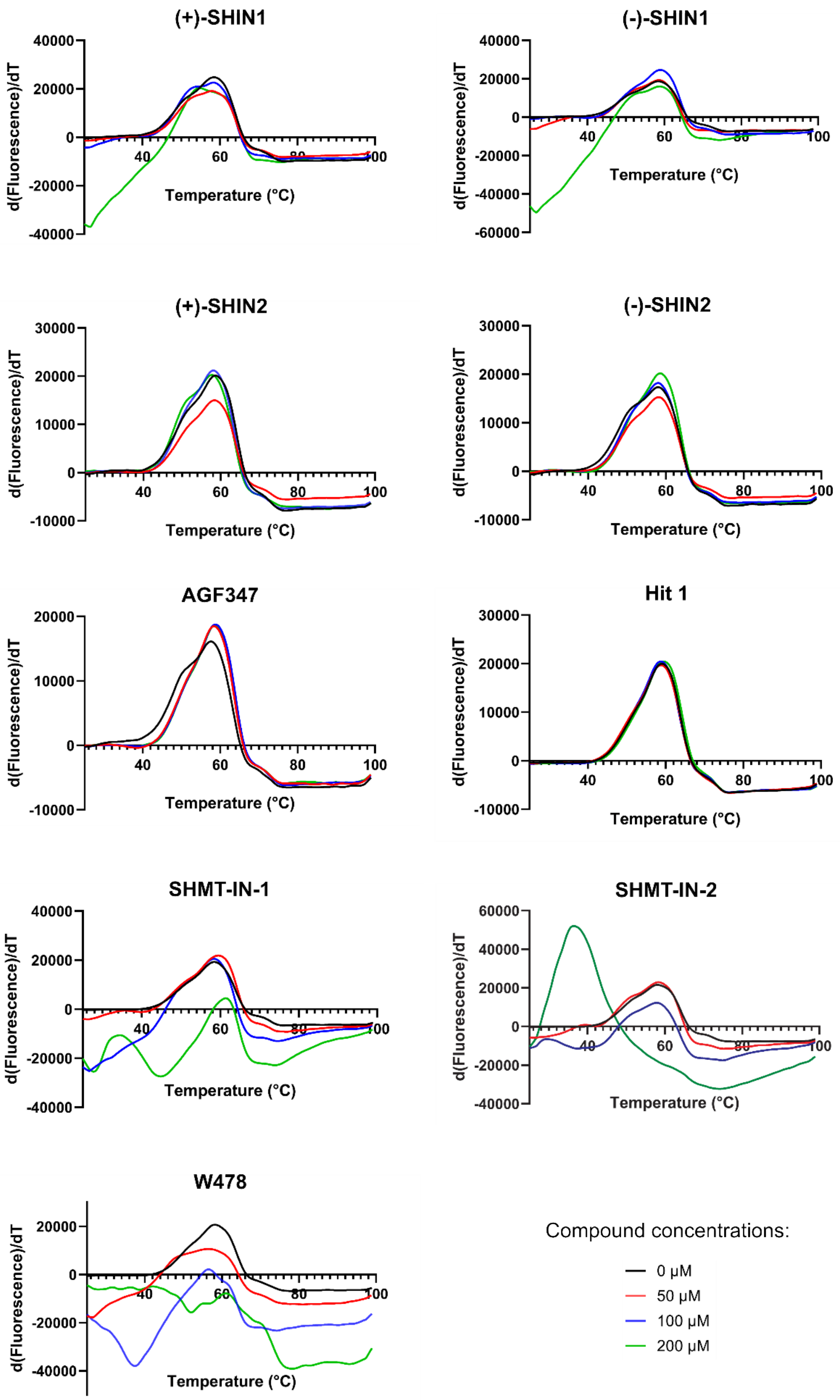

Figure S5: Binding of compounds to ADH as analyzed in protein thermal shift assays. (+)-SHIN1, (-)-SHIN1, (+)-SHIN2, (-)-SHIN2, AGF347, Hit 1, SHMT-IN-1, SHMT-IN-2 and W478 were applied at compound concentrations of 0 µM (black), 50 µM (red), 100 µM (blue) and 200 µM (green). All experiments were performed in triplicate.

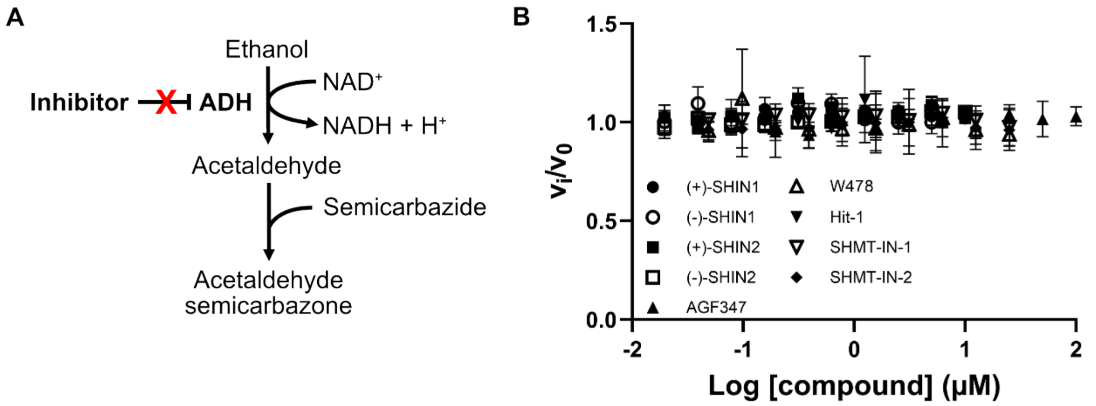

Figure S6: Activity of inhibitors against ADH. (A) Reaction of ADH. (B) Initial reaction rates v_i of compound-containing reactions normalized against the initial reaction rates v_0 of the control reactions are plotted against the compound concentrations. All experiments were performed at least in triplicate. Error bars represent standard deviations.

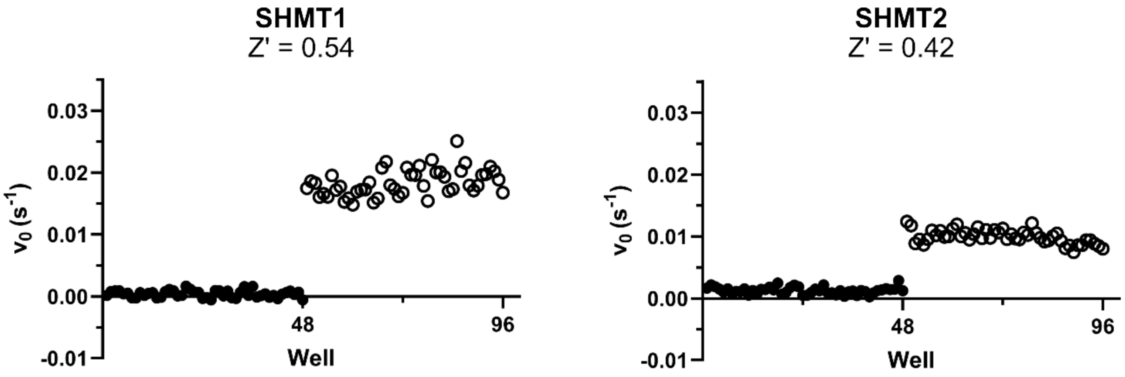

Figure S7: Determination of the Z´ factor of the SHMT1/2 inhibition assays. One representative experiment is shown for SHMT1 (left) and SHMT2 (right). Full circles represent negative controls and empty circles positive control reactions. The calculated Z´ factor for the SHMT1 assay is 0.52 ± 0.04 and the Z´ factor for the SHMT2 assay is 0.36 ± 0.05. Assays were performed in triplicate.

Supplementary Tables

Table S1: Compounds tested and corresponding chemical structures.

Compound	Structure
(+)-SHIN1	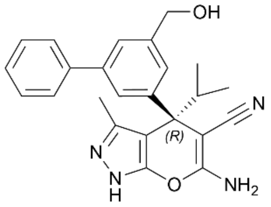
(-)-SHIN1	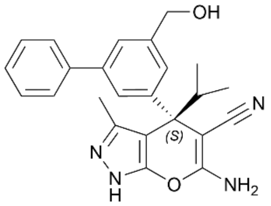
(+)-SHIN2	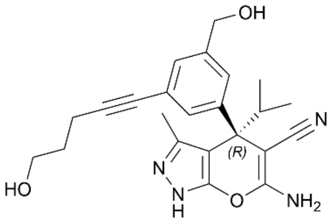
(-)-SHIN2	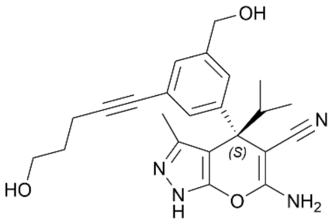
AGF347	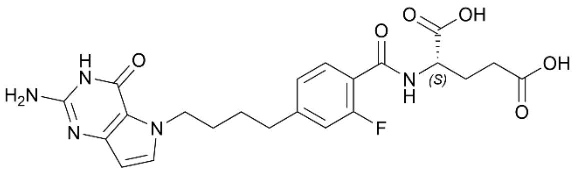
Hit 1	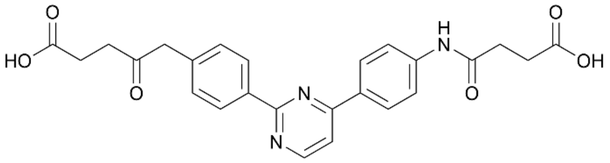

Compound	Structure
W478	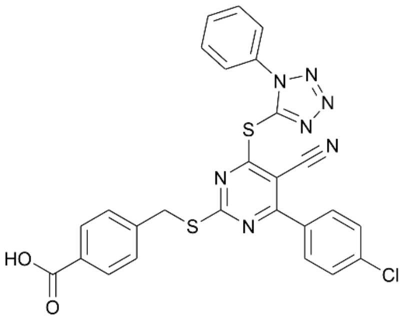
SHMT-IN-1	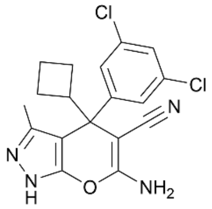
SHMT-IN-2	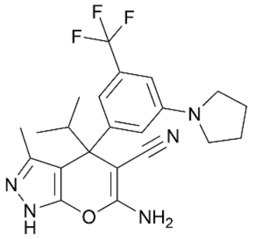

Table S2: Characterization of purified SHMT1 and SHMT2 (mean values ± standard deviations of three independent experiments).

	SHMT1	SHMT2
% Polydispersity *	4.5 ± 0.3	21.2 ± 0.9
Molecular weight (kDa) **	203 ± 51 ***	281 ± 119 ***
Specific activity (U/mg)	0.46 ± 0.01	0.42 ± 0.01
K_m L-allo-threonine (mM)	1.22 ± 0.40	3.99 ± 1.03

* Determined by dynamic light scattering (DLS)
** Determined by static light scattering (SLS)
*** Tetrameric protein, molecular weights of the monomeric SHMT1 and SHMT2 are 55.8 kDa and 56.1 kDa, respectively.

Table S3: Counter assays. Binding of test compounds to ADH as determined in thermal shift assays (ΔT_m are indicated at 100 µM compound concentration) and activities of test compounds against ADH. All experiments were carried out at least in triplicate (mean values ± standard deviations).

Compound	ADH ΔT_m (°C)	Activity of ADH (%) at the indicated concentration of compound
(+)-SHIN1	0.17 ± 0.22	102 ± 2.5 % at 10 µM
(-)-SHIN1	0.54 ± 0.34	103 ± 2.5 % at 10 µM
(+)-SHIN2	-0.87 ± 0.19	105 ± 1.5 % at 10 µM
(-)-SHIN2	-0.37 ± 0.22	105 ± 2 % at 10 µM
AGF347	0.15 ± 0.45	103 ± 5 % at 100 µM
Hit 1	-0.25 ± 0.05	102 ± 2 % at 10 µM
W478	-1.95 ± 0.83	94 ± 6 % at 25 µM
SHMT-IN-1	-0.44 ± 0.39	98 ± 10 % at 25 µM
SHMT-IN-2	0.17 ± 0.34	96 ± 10 % at 25 µM

Experimental Methods and Materials

The general chemicals were purchased from Carl Roth GmbH + Co. KG (Karlsruhe, Germany), VWR International GmbH (Darmstadt, Germany), Merck KGaA (Darmstadt, Germany), and Thermo Fisher Scientific (Life Technologies GmbH, Darmstadt, Germany). The supplier for more specific consumables is stated in the method description itself below.

Plasmid construction and recombinant protein expression

The protein-coding sequence of human *SHMT1* (NCBI reference sequence: NM_004169.5:199-1650) was amplified from cDNA. Using Gateway® Technology, the CDS was integrated into the expression vector pDEST17 (Thermo Fisher Scientific, 11803012) for the expression of a 6xHis fusion protein. The expression clones were sequence verified by the Sanger sequencing service of GENEWIZ Germany GmbH (Leipzig, Germany). A codon-optimized version of the human *SHMT2* (NM_001166357.1:332-1783) for bacterial expression was synthesized by the GeneArt gene synthesis service (Thermo Fisher Scientific). The delivered CDS in pENTR221 was transferred into pDEST17 for the expression of a 6xHis fusion protein.

The expression plasmid for 6xHis-SHMT1 was transformed into One Shot™ BL21(DE3)pLysE Chemically Competent *E. coli* (Thermo Fisher Scientific, C656503). Bacterial cultures were grown in LB medium containing 50 µg/ml carbenicillin and 0.01 % (v/v) antifoam 204 (Merck, A6426) at 37 °C. When OD_{600} reached 0.4-0.6 temperature was adjusted to 16 °C and protein expression induced with 0.4 mM IPTG. The expression plasmid for 6xHis-SHMT2 was transformed into One Shot™ BL21(DE3)pLysE Chemically Competent *E. coli*. Bacterial cultures were grown in TB medium containing 50 µg/ml carbenicillin and 0.01 % v/v antifoam 204 at 37 °C. When OD_{600} reached 0.5-0.7 temperature was adjusted to 18 °C and protein expression induced with 0.4 mM IPTG. Biomass was collected 16-18 h after induction by centrifugation at 4000 x *g* for 20-30 min at 4 °C and stored at -80 °C for up to two months.

Sequence of expressed 6xHis-SHMT1

MSYYHHHHHHLESTSLYKKAGFMTMPVNGAHKDADLWSSHDKMLAQPLKDSDVEVYNIIKKESNRQRVGLELIASENFASRAVLEALGSCLN
NKYSEGYPGQRYYGGTEFIDELETLCQKRALQAYKLDPQCWGVNVQPYSGSPANFAVYTALVEPHGRIMGLDLPDGGHLTHGFMTDKKKISA
TSIFFESMPYKVNPDTGYINYDQLEENARLFHPKLIIAGTSCYSRNLEYARLRKIADENGAYLMADMAHISGLVAAGVVPSPFEHCHVVTTTTHK
TLRGCRAGMIFYRKGVKSVDPKTGKEILYNLESLINSAVFPGLQGGPHNHAIAGVAVALKQAMTLEFKVYQHQVVANCRALSEALTELGYKIVT
GGSDNHLILVDLRSKGTDGGRAEKVLEACSIACNKNTCPGDRSALRPSGLRLGTPALTSRGLLEKDFQKVAHFIHRGIELTLQIQSDTGVRATLKE
FKERLAGDKYQAAVQALREEVESFASLFPLPGLPDF*

Sequence of expressed 6xHis-SHMT2

MSYYHHHHHHLESTSLYKKAGFMAIRAQHSNAAQTQTGEANRGWTGQESLSDSDPEMWELLQREKDRQCRGLELIASENFCSRAALEALGS
CLNNKYSEGYPGKRYYGGAEVVDEIELLCQRRALEAFDLDPAQWGVNVQPYSGSPANLAVYTALLQPHDRIMGLDLPDGGHLTHGYMSDVK
RISATSIFFESMPYKLNPKTGLIDYNQLALTARLFRPRLIIAGTSAYARLIDYARMREVCDEVKAHLLADMAHISGLVAAKVIPSPFKHADIVTTTT
HKTLRGARSGLIFYRKGVKAVDPKTGREIPYTFEDRINFAVFPSLQGGPHNHAIAAVAVALKQACTPMFREYSLQVLKNARAMADALLERGYSL
VSGGTDNHLVLVDLRPKGLDGARAERVLELVSITANKNTCPGDRSAITPGGLRLGAPALTSRQFREDDFRRVVDFIDEGVNIGLEVKSKTAKLQ
DFKSFLLKDSETSQRLANLRQRVEQFARAFPMPGFDEH*

Protein purification

E. coli cell pellets of SHMT1 expressions were resuspended in extraction buffer (50 mM HEPES pH 7.4, 300 mM NaCl, 20 mM imidazole (BioUltra, ≥99.5 % (GC), Merck, 56749), 5 % glycerol, 2 mM $MgCl_2$), 8 ml / g wet weight. 2 mM DTT, 0.2 mM pyridoxal phosphate (PLP, Carl Roth, 1T9H), EDTA-free protease inhibitor (cOmplete™ ULTRA-Tablets, Merck, 5892953001 or 1 mM PMSF), 2 mg/ml lysozyme and Benzonase® Nuclease (5 U/ml, Merck, 70746) were added to the cell suspension and incubated on a horizontal shaker for 30-45 min at room temperature. After that, the cell lysate preparation was cooled down and sonicated on ice. After centrifugation at 15,000 x *g* for 30 min, the protein lysate was diluted with 1 volume buffer A (50 mM HEPES pH 7.4, 300 mM NaCl, 20 mM imidazole, 5 % glycerol, 1 mM DTT) and load onto 1 ml HiTrap TALON® crude column (Cytiva, 28953766) connected to an ÄKTA pure™ 25 M (Cytiva). The target protein was eluted with 75 % B (188 mM imidazole). Elution fractions were pooled and desalted by dialysis (50 mM HEPES pH 7.5, 100 mM NaCl, 0.5 mM EDTA, 5 % glycerol, 0.5 mM DTT).

SHMT1 protein solution was dialyzed using a 3.5 MWCO tubing (dialysis membrane Spectra/Por® 7 MWCO 3500, Carl Roth, E859) overnight for 20 h in desalting buffer (50 mM HEPES pH 7.5, 100 mM NaCl, 0.5 mM EDTA, 5 % glycerol) and another 4 h in fresh buffer at room temperature. This protein preparation was then concentrated to a protein stock concentration of 10-20 µM tetrameric SHMT1 using Amicon® Ultra centrifugal filter units (30 kDa MWCO, Merck, UFC903024 & UFC803024). The concentration of 6xHis-SHMT1 was determined by UV/Vis using the Implen NanoPhotometer® NP80 (IMPLEN, Munich, Germany). Protein stock preparations were aliquoted and flash-frozen in liquid nitrogen before storing at -80 °C. The purified protein as well as fractions collected during the purification were analyzed by SDS-PAGE and western blotting (data not shown).

Preparation of 6xHis-SHMT2 protein stocks was done with the same procedure. Slightly different buffer compositions were used for A (50 mM HEPES pH 7.4, 300 mM NaCl, 100 mM KCl, 20 mM imidazole, 5 % glycerol, 1 mM DTT), B (50 mM HEPES pH 7.4, 300 mM NaCl, 100 mM KCl, 250 mM imidazole, 5 % glycerol, 1 mM DTT) and the desalting buffer (50 mM HEPES pH 7.5, 50 mM NaCl, 50 mM KCl, 0.5mM EDTA, 5 % glycerol, 0.5 mM DTT). The elution from affinity column was isocratic at 75 % B.

Static light scattering

Static light scattering experiments were performed using the Litesizer 500 (Anton Paar, Germany). All solutions were filtered prior to the measurement. Proteins were applied in 50 mM HEPES pH 7.5, 100 mM NaCl, 0.5 mM EDTA, 0.005 % (v/v) Tween 20, 1 mM DTT and 5 % (v/v) glycerol as a concentration row comprising three different concentrations starting with the stock concentration diluted 1:2 with buffer (SHMT1 stock concentrations applied were 4.77 mg/ml, 3.89 mg/ml or 1.78 mg/ml, depending on the specific protein batch applied for three independent experiments; SHMT2 stock concentrations applied were 2.35 mg/ml, 2.42 mg/ml and 1.10 mg/ml, depending on the specific protein batch applied for three independent experiments). Toluol was used as scattering reference. Measurements were carried out at 20 °C in low volume quartz cuvettes. Results of each independent experiment reflected the average of 50 runs 10 seconds each.

Dynamic light scattering

Dynamic light scattering experiments were performed using the Litesizer 500 (Anton Paar, Germany). Proteins were applied at their undiluted stock concentration (SHMT1: 21.4 µM, 17.0 µM or 15.7 µM,

depending on the specific protein batch applied for three independent experiments; SHMT2: 10.4 µM or 10.7 µM, depending on the specific protein batch applied for three independent experiments) in 50 mM HEPES pH 7.5, 100 mM NaCl, 0.5 mM EDTA, 0.005 % (v/v) Tween 20, 1 mM DTT and 5 % (v/v) glycerol. Measurements were carried out at 20 °C in low volume quartz cuvettes. Results of each independent experiment reflected the average of 100 runs 10 seconds each.

Enzymatic activity assay

Assays were carried out in assay buffer (50 mM HEPES pH 7.5, 100 mM NaCl, 0.5 mM EDTA, 0.005 % (v/v) Tween 20 and 1 mM DTT) supplemented with 2 % DMSO at room temperature. Reaction mixtures were composed of 0.5 µM SHMT1 or SHMT2, 2 mM L-allo-threonine (Thermo Fisher Scientific, 198542500), 1 mM NADH (Thermo Fisher Scientific, J61461.03) and 1 U of the coupling enzyme alcohol dehydrogenase (ADH, Sigma-Aldrich, A7011). The reaction was started by the addition of L-allo-threonine and the absorbance at 340 nm was measured after 18 minutes. A control reaction without any enzyme was included. The specific activity was calculated in U/mg, where 1 U is defined as the conversion of 1 µmol L-allo-threonine and 1 µmol NADH per minute.

Determination of the K_m value of L-allo-threonine

The K_m value of L-allo-threonine for SHMT1 and SHMT2 was determined in assay buffer (50 mM HEPES pH 7.5, 100 mM NaCl, 0.5 mM EDTA, 0.005 % (v/v) Tween 20 and 1 mM DTT) supplemented with 2 % DMSO at room temperature applying a dilution row of L-allo-threonine. The reaction was composed of 0.0195-10 mM L-allo-threonine (Thermo Fisher Scientific, 198542500), 0.1 mM NADH (Thermo Fisher Scientific, J61461.03), 10 U ADH (Sigma Aldrich, A7011) and 0.5 µM SHMT1 or 1.5 µM SHMT2. The substrate conversion was monitored by recording the absorbance at 340 nm (consumption of NADH) at room temperature in transparent 96-well plates (Corning, 3596) with a total reaction volume of 100 µl per well using the SPARK plate reader (Tecan). The initial reaction velocity was determined by linear regression of the first 30 seconds and plotted against the concentration of L-allo-threonine. The K_m value was determined by nonlinear regression applying the Michalis-Menten equation ($Y=V_{max}*X/(K_m+X)$).

Determination of the enzymatic activity of ADH in the presence of test compounds

The oxidation of ethanol by ADH was measured under the influence of increasing test compound concentrations. All assays were carried out in assay buffer (50 mM HEPES pH 7.5, 100 mM NaCl, 0.5 mM EDTA, 0.005 % (v/v) Tween 20 and 1 mM DTT) at room temperature. Reaction mixtures contained 10 U ADH, 380 mM ethanol, 2 mM NAD^+, 50 mM semicarbazide and varying concentrations of test compounds (1.526 nM – 100 µM, depending on the compound). Dilution series of test compounds in DMSO or DMSO only (control) were pipetted into a transparent 96-well plate. A master mix composed of NAD^+ and ADH was added to the wells. The reaction was started by the addition of assay buffer supplemented with the respective concentration of ethanol to a total reaction volume of 100 µl. The absorbance at 340 nm was recorded in transparent 96-well plates. The final DMSO concentration within the assay was 2 %. The initial reaction velocities v_i for each inhibitor concentration were determined by linear regression of the first 30 seconds and normalized against the initial reaction velocity of the non-inhibited reaction v_0. v_i/v_0 values were plotted against the inhibitor concentration and the IC_{50} values were determined by applying a nonlinear four-parameter regression ($Y=Bottom + (Top-Bottom)/(1+10^((LogIC_{50}-X)*HillSlope))$).

Absorbance scan of test compounds

The absorbance spectra in the wavelength range of 200–1000 nm of all test compounds were recorded at room temperature in assay buffer with a total volume of 100 µl. The compound concentrations corresponded to the highest test concentration in the inhibition assays ((+)-SHIN1: 10 µM, (-)-SHIN1: 10 µM, (+)-SHIN2: 10 µM, (-)-SHIN2: 10 µM, AGF347: 100 µM, SHMT-IN-1: 25 µM, SHMT-IN-2: 25 µM, Hit 1: 10 µM, W478: 25 µM).

Z´ factor determination and calculation

DMSO and threonine were added to the wells and the reaction was started by the addition of a master mix composed of NADH, both enzymes (SHMT1 or SHMT2 and coupling enzyme ADH) and buffer using a multi-channel pipette. The absorbance at 340 nm was recorded in transparent 96-well plates with a total reaction volume of 100 µl per well.

The Z´ factor was calculated using the equation $Z' = 1 - (3 \times SD_{PC} + 3 \times SD_{NC})/(v_{0,PC} - v_{0,NC})$, where SD is standard deviation and v_0 is the initial reaction rate as mean value of 48 wells. PC is the positive control reaction (0.5 µM SHMT1 or 1.5 µM SHMT2, 2 mM L-allo-threonine, 0.1 mM NADH, 10 U alcohol dehydrogenase and 2 % (v/v) DMSO) and NC is the negative control reaction (2 mM L-allo-threonine, 0.1 mM NADH and 2 % (v/v) DMSO).
